# Personality Functioning Cannot Only Be One Thing: A Call for Theoretical Pluralism

**DOI:** 10.1002/pmh.70085

**Published:** 2026-06-09

**Authors:** Orestis Zavlis

**Affiliations:** ^1^ Psychoanalysis Unit University College London London UK

## Abstract

Since its origins in recent diagnostic perspectives, the concept of personality functioning has been exclusively defined as a general factor of personality (pathology). Researchers have justified this definition by appealing to descriptive empirical findings (e.g., positive correlations among various personality disorder indicators) or traditional psychoanalytic theories (e.g., ego psychology). In this review, I argue that this monopoly of interpretive viewpoints is currently impeding progress in the field of personality functioning. To begin with, I review seminal psychoanalytic theories and show how all of them run contrary to the view of personality functioning as a ‘single, underlying capacity’. Subsequently, I review the empirical literature on personality functioning and argue that this construct could be interpreted in either a descriptive way (in which case it is theoretically empty) or an explanatory way (in which case it is fundamentally incompatible with the kinds of psychoanalytic theories typically invoked to justify it). Finally, I present three alternative ways of conceptualising personality functioning (viz., a configurational way, a dialectic way and a relational way) and conclude the paper by suggesting that the future of this line of inquiry lies in formally articulating and empirically scrutinising as many such competing theoretical viewpoints as possible.

## 1 Introduction

Since its introduction in the DSM‐5 AMPD, the idea of personality functioning has sparked heated debate about the definition and structure of personality pathology. Most of this debate has thus far operated at the empirical level, focusing on the psychometric overlap of personality functioning with (maladaptive) personality traits (García et al. 2024; Morey 2019; Morey et al. 2022; Sleep et al. 2019, 2020; Sleep and Lynam 2022; Widiger and Smith 2025) or general indicators of psychopathology (Bender 2019; McCabe et al. 2022; Oltmanns et al. 2018; Widiger et al. 2019). At the same time, however, this empirical debate has been accompanied by a form of theoretical foreclosure, whereby questions about the basic definition of personality functioning have been treated as settled rather than open to contestation (e.g., Gilbert 2026; Hopwood 2025; Zavlis 2025a, 2025b; Zimmermann et al. 2025). Consequently, the construct of personality functioning has become difficult to reject or revise in light of conflicting empirical evidence, because its core assumptions have been protected at the level of definition rather than exposed to empirical examination.

In this paper, I argue that progress regarding the concept of ‘personality functioning’ can only be achieved by embracing theoretical pluralism: that is, by recognising that personality functioning can be conceptualised in various theoretically distinct ways, rather than by being restricted to a single ‘unitary’ perspective. To make this case, I structure the paper as follows. First, I argue that the theories invoked to justify the definition of personality functioning as a unitary entity run, in fact, contrary to this perspective because they typically posit various developmental mechanisms rather than a single developmental capacity. Second, I show that personality functioning, as currently operationalised, can be understood either as a descriptive index of severity (in which case it is theoretically empty) or as a common‐cause mechanism (in which case it runs contrary to most, if not all, psychodynamic theories). Third, I illustrate how ‘personality functioning’ can be formally defined in at least three alternative ways: namely, in a configurational way, a dialectical way and a relational way (following seminal theories by Kernberg, Blatt and Fonagy, respectively). Finally, I propose that the field can only make progress by explicitly articulating and empirically testing these competing representations of personality functioning, rather than by assuming (via definitional fiat) that personality functioning must only be defined as a ‘unitary capacity’.

## 2 A Brief History of Psychoanalytic Theories on Personality

To begin with, it is worth substantiating the claim that existing psychodynamic theories do not support a ‘unitary’ conception of personality functioning. This is an important claim to substantiate, as there has been a trend in the field to invoke at least six major families of psychodynamic theory to retroactively justify the view that personality functioning reflects a single capacity (e.g., Gilbert 2026; Hopwood 2025). In what follows, however, I show that this interpretation rests on a systematic conflation of distinct developmental mechanisms drawn from these theories.

First, let us begin with classic Freudian psychoanalysis. Under Freud, ‘development’ was understood in terms of various conflicting processes, not in terms of a single ‘maturation process’ as recent theorists have posited (e.g., Hopwood 2025). To briefly illustrate, Freud's early drive model already posited multiple developmental processes, including *psychosexual development*, *the formation of character* and *the emergence of defensive processes* (Freud 1905/1953, 1926/1959, 1923/1961). Subsequently, with the introduction of his structural model, Freud further differentiated personality in terms of three interacting systems involved with *biological pressures* (the id), *self‐regulation* (the ego) and *moral evaluation* (the superego) (Freud 1923/1961). Finally, Freud repeatedly emphasised that psychopathology could arise from conflict, fixation or regression at different points within and across these systems, rather than from a uniform deficit in development (Freud 1917/1957, 1926/1959). Under Freud, then, psychological development never involved ‘only one process’ but instead involved a host of interacting developmental systems.

Second, the next major family of psychodynamic theory was ego psychology, which emerged in the 1930s (e.g., Freud 1936/1966; Hartmann 1958; Hartmann et al. 1947). During this time, ego psychologists increasingly focused on how individuals adapt to reality through the development of various ‘ego’ functions, including *reality testing*, *affect regulation*, *impulse control* and *defensive organisation* (Hartmann 1958; Kernberg 1975; Rapaport 1951; Vaillant 1992). Development within this tradition was indeed often described along a single line of maturation, commonly referred to as ‘ego strength’ (Hartmann 1958; Loevinger 1976; Vaillant 2012). Crucially, however, this developmental line was explicitly limited in scope: That is, ‘ego strength’ was never equivalent to the contemporary concept of ‘personality functioning’; instead, ‘ego strength’ was restricted to a circumscribed set of self‐regulatory capacities like the management of anxiety, conflict and external demands on the self by reality (Blatt 2008; Freud 1936/1966; Hartmann 1958; Kernberg 2016; Vaillant 2012). Importantly, this distinction held (and arguably became even more salient) in later developmental theories, like the ones from Erikson (1963) and Loevinger (1976), who described the development of specific self‐capacities (e.g., the development of autonomy, identity, meaning‐making or other self‐reflective capacities), which are only one subset of the development of broad personality capacities (e.g., broad ways of ‘thinking, feeling, fantasising, desiring, fearing, coping, defending, attaching, relating and experiencing self and others’; e.g., Shedler 2022; Shedler and Westen 2004; Widiger 2011; Zavlis 2024). Thus, ego psychology was never a theory of personality functioning writ large but rather a theory of the development of specific self‐regulatory capacities.

Third, in response to this limited scope of ego psychology, the next major psychodynamic theory was, historically, object relations—so‐called because it focused not on a single object (e.g., the ‘ego’ or the ‘self’) but rather on the relations among various objects (usually, humans; sometimes, specific body parts; and other times, physical objects like transitional objects) (Fairbairn 1941; Kernberg 1976; Klein 1946; Laplanche and Pontalis 2018; Winnicott 1951). Beginning in the 1940s, object relations theorists departed from both Freudian and ego psychology by instead suggesting that ‘people do not seek drive satisfaction so much as they seek relationships’ (MacWilliams 2020, 48, Chapter 2). Development, from this perspective, was inherently relational: It involved the capacity to internalise stable representations of others (e.g., Klein 1946; Mahler et al. 1975; Winnicott 1960), to experience self and other as separate yet emotionally connected (e.g., Mahler et al. 1975; Winnicott 1965) and to tolerate the uncertainty that is inherent in all relationships (see Bion 1962; Klein 1940). Psychopathology, in this sense, was also relational: It involved maladaptive ways of positioning the self in relation to others (e.g., split and/or unstable representations of self and other, primitive anxieties around closeness vs. separation and rigid expectations from others), which operated either in fantasy or in actual relationships (Fairbairn 1952; Kernberg 1975; Klein 1946). Importantly, disturbances in this (object) relational functioning were not understood as secondary consequences of impaired ‘ego development’ but rather as expressions of a distinct psychological system shaped by early relational experience (Fairbairn 1954; Kernberg 1976; MacWilliams 2020; Pine 1990). Object relations theory, therefore, was fundamentally distinct from ego psychology in that it focused less on the development of ‘self‐capacities’ and more on the development of adaptive ways of relating to others (and, by extension, adaptive ways of relating to the self).

Fourth, in light of these theoretical differences (that focus too much on either the self or others), an effort was made to integrate the self‐versus‐other developmental lines, with Kernberg achieving this most successfully in his structural model of personality (Kernberg 1967, 1970, 1975). Importantly, although ego and object relations were unified under a single framework, the framework itself was by no means unidimensional, as it did not involve a ‘single’ capacity but rather qualitatively distinct configurations of various such capacities (initially, identity diffusion, defensive organisation and reality testing; subsequently, object relations, aggression and levels of instinctual fixation; see Kernberg 1975). To illustrate, a neurotic configuration (or organisation), in Kernberg's framework, involves a pattern in which identity is relatively well‐integrated, object relations are stable, and conflict is managed through relatively mature defences (like repression or intellectualisation) (Kernberg 1970). By contrast, a borderline configuration (or organisation) involves a pattern in which identity is diffuse (i.e., fluid or unintegrated), object relations are unstable, and defensive operations are organised around primitive mechanisms like splitting (‘black‐and‐white’ thinking) and projection (i.e., misattributing personal qualities to others; Kernberg 1967, 1984). Finally, a psychotic configuration involves a pattern in which identity is severely ‘disintegrated’, object relations are fragmented or poorly differentiated from reality, and reality testing itself is ‘completely lost or absent’ (Kernberg 1970, 810). Importantly, and as I further argue subsequently, these configurations are fundamentally distinct from the currently unitary definition of personality functioning because they do not treat self–interpersonal impairments as interchangeable indicators of a single latent vulnerability but rather as latent configurations representing distinct vulnerabilities (i.e., neurotic, vs. psychotic, vs. borderline vulnerabilities; more on this in Section 3).

Fifth, and importantly, this distinction (between self‐functioning and social functioning) was made even more explicit in dialectical models of personality, such as the one proposed by Blatt (2008). Under Blatt's model, personality development does not involve a ‘single underlying capacity’ (e.g., Gilbert 2026, 3), because it involves, to use his own words, ‘a dialectic synergistic interaction between the development of capacities for interpersonal relatedness and the development of self‐definition or identity’ (which roughly corresponds to object relations vs. ego lines of development; Blatt and Luyten 2009, 793). Under this model, mental health arises when these two developmental lines balance and mutually support each other (see Blatt and Luyten 2009; Blatt and Zuroff 1992; Luyten and Blatt 2013). Mental illness, by contrast, emerges ‘from an excessive emphasis on one developmental line and the defensive avoidance of the other’ (see Luyten et al. 2017, 179), such that specific illnesses involve primarily *self‐focused problems* (e.g., introjective, perfectionistic and narcissistic problems involving excessive self‐criticism; Blatt 1995) or *interpersonally focused problems* (e.g., anaclitic, dependent or borderline problems; Blatt 1992). Once again, then, we see that personality and its development are not reduced to a ‘one process’ model but are rather viewed as a dynamic system in which self‐functioning and interpersonal functioning interact to generate qualitatively distinct patterns of psychopathology.

Finally, we reach one of the most recent and seminal psychodynamic accounts of personality, namely, the ‘mentalising’ approach to personality and psychopathology (see Luyten et al. 2020). Although ‘mentalising’ (defined as the capacity to conceive oneself and others in terms of intentional mental states; Fonagy et al. 2018) has been suggested to reflect a ‘single capacity’ (Gilbert 2026, 3), from a mechanistic viewpoint, both mentalising and related capacities (e.g., attachment and epistemic trust) are inherently multidimensional and context‐sensitive (e.g., see Zavlis, Story, et al. 2025; Zavlis et al. 2026; Zavlis and Fonagy 2024a). Indeed, in a recent systematic review on the measurement of mentalising, the main theorists of mentalising have bemoaned the persistent tendency to treat reflective (aka ‘mentalising’) functioning as a unitary entity by arguing that ‘mentalising’ comprises partially independent processes (e.g., affective vs. cognitive; automatic vs. controlled; and self‐focused vs. other‐focused) that operate differently across relational contexts (see Hawksdale et al. 2025, 13). Moreover, in computational models of mentalising and related capacities, the core mechanism is explicitly framed not as an underlying ‘trait‐like’ deficit that operates invariantly across life settings but rather as a process of ‘relational inference’: that is, making sense of oneself and others in ‘particular attachment contexts’ (see Zavlis, Story, et al. 2025; Zavlis et al. 2026; Zavlis and Fonagy 2024a). In that sense, although mentalising can be understood as a transdiagnostic and developmental capacity, it is by no means unitary because it can fail in many distinct ways and across distinct relational settings (see Fonagy et al. 2015; Fonagy and Allison 2023; Fonagy and Luyten 2009, 2018; Luyten and Fonagy 2015).

Based on this brief review above, we can clearly see that there is little theoretical basis for a unitary conception of ‘personality functioning’ within the psychodynamic tradition. Indeed, most psychodynamic theories appear to clearly distinguish between multiple developmental mechanisms rather than collapse all of them into a single ‘capacity’. In the next section, I solidify this thesis by showing precisely how the current conception of personality functioning commits it to either a descriptive ontology (in which case it renders it theoretically empty) or a mechanistic ontology (in which case it renders it fundamentally nonanalogous to these major psychodynamic theories).

## 3 Personality Functioning: Descriptive or Explanatory?

To understand why personality functioning, as currently operationalised, differs fundamentally from major psychodynamic theories, it is instructive to revisit the construct's origins. On this, it must be recognised that the construct of ‘personality functioning’ did not emerge as the logical endpoint of ‘psychodynamic theory’ as recent theorists have implied (e.g., Gilbert 2026; Hopwood 2025; Zimmermann et al. 2025). Instead, the construct of ‘personality functioning’ was specifically created as a diagnostic marker of ‘personality severity’ by Bender et al. (2014).

Indeed, Bender et al. (2014) were explicit that their primary aim was more pragmatic than theoretical: Specifically, their aim was to construct ‘a scale of severity of impairment in personality functioning’, which would allow clinicians to rate ‘not only the existence of personality psychopathology but also its severity’ (see Bender et al. 2014, 332). Importantly, the authors achieved this by focusing on the ‘robust elements’ from clinician‐rated measures on personality pathology, concluding that it is ‘indeed, possible to delineate a global coherent dimension of personality pathology’ (see Bender et al. 2014, 339). This process resulted in the creation of a psychometric scale (viz., the Level of Personality Functioning Scale [LPFS]), which comprised four facets (identity, self‐direction, empathy and intimacy), two higher order domains (self‐functioning and interpersonal functioning) and a global dimension of severity that formed the basis for the assessment of personality disorder in the DSM‐5 AMPD.

From this process, it is clear that ‘personality functioning’, as a scientific entity, was first and foremost a descriptive entity. That is, the aim of personality functioning was to summarise the main impairments of ‘personality disorder’, not to explicitly formalise a mechanism through which those impairments emerge in the first place (Zavlis et al. 2026). Importantly, this is analogous to related descriptive constructs in psychology, such as the ‘general factor of personality’ and the ‘general factor of psychopathology’, both of which correlate with ‘personality functioning’ almost perfectly, likely because they all simply quantify the burden of various psychological problems (e.g., see Bender 2019; McCabe et al. 2022; Oltmanns et al. 2018; Widiger et al. 2019; Zavlis and Fonagy 2026). In this way, the main operationalisation of personality functioning can be understood as descriptive because it is simply a summary of various domains of functioning, rather than a mechanistic theory of personality (e.g., see Fried 2020; Fried et al. 2021).

Nevertheless, although ‘personality functioning’ has been, and continues to be, operationalised in a descriptive way, the statistical methods by which this description happens carry with them at least one substantive tenet: namely, the view of ‘personality functioning’ as a common cause of various personality indicators (Borsboom 2005; Borsboom et al. 2003; van Bork et al. 2017). To briefly elaborate, consider for a moment the kinds of statistical models (viz., reflective latent variable models) that are currently used to operationalise personality functioning. According to these models, covariation among various personality items is assumed to be caused by a single latent entity (here, ‘personality functioning’; see Borsboom 2005 for a detailed conceptual analysis). Importantly, this assumption can be expressed differently depending on how the latent variable model is operationalised, with the common‐cause entity ‘causing’ individual items (e.g., ‘I often have difficulty understanding the thoughts and feelings of others.’), facets (e.g., self‐direction vs. intimacy) or higher order domains (i.e., self‐functioning vs. social functioning) (Dolan and Borsboom 2023). In any case, regardless of the specific modelling details, the common‐cause assumption remains invariant across these latent descriptive models, suggesting that if one wishes to interpret the current operationalisation of personality functioning in a substantive way, one would have to use this common‐cause way of viewing it (see Borsboom et al. 2003).

However, based on our previous review, it does not appear that any psychodynamic theory suggests that personality functioning can be reduced to such a ‘single process’ common‐cause entity. Indeed, as reviewed previously, psychodynamic theories differ in the degree to which they privilege self‐functioning (‘ego maturity’; Hartmann 1958), versus relational functioning (‘object relations’; Fairbairn 1954), versus a specific configuration thereof (a ‘personality organisation’; Kernberg 1970), versus a dialectic thereof (‘self‐polarity vs. interpersonal polarity’; Blatt 2008), versus an affective, cognitive, internally based or automatic aspect of self–other ‘reflective’ functioning (Fonagy et al. 2018). What is clear, however, is that none of these psychodynamic theories collapse these various personality mechanisms into a common‐cause vulnerability.

This mismatch implies that personality functioning, as currently operationalised, does not reflect any psychodynamic theory when it is interpreted substantively. Importantly, this conflict only exists when someone adopts an explanatory (aka substantive) view of personality functioning. Indeed, if one adopts a purely descriptive view of personality functioning, then no such conflict exists: No specific developmental or theoretical claims follow from it because the main role of the construct is to summarise various impairments, not to posit an explanatory process of how said impairments arose in the first place (e.g., see Sharp 2022; Tyrer et al. 2011; Watts et al. 2023).

Together, then, these considerations suggest that if one commits to interpreting personality functioning in an explanatory (rather than descriptive) way, then one must also accept at least two logical consequences from this commitment: first, that the (data‐generating) mechanisms of the typical models used to operationalise ‘personality functioning’ do not match any major psychodynamic theories; second, that even if these mechanisms did match a particular theory, other theories would be left as alternatives. This second point is worth emphasising: Even if personality functioning was currently operationalised in a theoretically rich way that matches a particular psychodynamic perspective, this operationalisation of personality functioning would not be exclusive because there are at least a handful of other theoretical perspectives that make different ontological and developmental commitments regarding the idea of ‘personality functioning’.

Although various theorists have recently proposed an alternative viewpoint (viz., that there is only ‘one way’ of interpreting personality functioning; e.g., Gilbert 2026; Hopwood 2025), in the next section, I advance my counterclaim, which suggests that personality functioning can be interpreted in at least three theoretically distinct ways.

## 4 Alternative Conceptualisations of Personality Functioning

In this section, I suggest that ‘personality functioning’ can be interpreted in at least three alternative ways: namely, a structural way, a dialectic way and a relational way. Crucially, I only outline three alternative conceptualisations of personality functioning for the purposes of illustration. Indeed, there can be many more alternative theoretical viewpoints, like behavioural viewpoints, which make radically different assumptions than psychoanalytic viewpoints (e.g., Kaiser 2026). However, given that the focus of this essay is to illustrate how psychoanalytic theories differ vastly in their interpretation of personality, what follows is a brief exploration of three such theories and the differing ways in which they define the ontology of personality and its functioning.

To begin with, let us first consider Kernberg's (1967, 1970, 1975, 1984) structural model of personality. As explained previously, Kernberg (1967, 1970, 1975, 1984) views personality in terms of three kinds of organisations: namely, the neurotic, borderline and psychotic organisations (see also Kernberg 2016). Each organisation reflects a *qualitatively distinct configuration* of key personality functions, including identity integration, defensive organisation and reality testing. Based on this theoretical exposition, my argument is that ‘personality functioning’, from Kernberg's viewpoint, cannot be understood as a general latent factor of these (and related) personality capacities. Instead, personality functioning from Kernberg's viewpoint can be more appropriately understood in terms of three interdependent, yet distinct, latent distributions (or organisations) of these personality variables (Zavlis 2026b).

To illustrate, if one were to operationalise Kernberg's theory using contemporary measures of personality, one would not simply summarise variation across these measures of personality and call the ensuing broad summary ‘personality functioning’. Instead, one would likely conduct some kind of mixture modelling to determine whether variation across key personality dimensions can be distilled into three distinct *latent distributions* (aka ‘organisations’ or ‘configurations’): namely, a neurotic configuration (scoring low on identity disintegration, primitive defences and reality testing), a psychotic configuration (scoring high on identity disintegration, primitive defences and reality testing) and a borderline configuration (scoring somewhat high on identity disintegration and primitive defences but low reality testing) (see Zavlis 2026b for a formal model on Kernberg's theory). Indeed, as other theorists have also suggested (e.g., Sharp and Wall 2021, 326), the current interpretation of ‘personality functioning’ as a general factor of personality (as well as psychopathology) makes little sense from Kernberg's viewpoint, because from his viewpoint, the kind of personality functioning that is directly related to personality pathology is the borderline kind of functioning (which is qualitatively distinct from and sits between the neurotic and psychotic levels of functioning; see also Zavlis 2026b). In that sense, a proper definition of personality disorders, from Kernberg's viewpoint, is not in terms of low scores on a general personality (and, by extension, psychopathology) factor but rather in terms of low scores on a more specific latent distribution (i.e., organisation of variables) that reflects ‘borderline’ kinds of difficulties in identity, defences and reality testing (and related personality domains).

Moving on, let us next consider Blatt's (2008) dialectical model of personality. As outlined previously, Blatt's (2008) model was explicitly and unambiguously two‐dimensional in the sense that it involved a dialectic between two personality capacities: self‐definition and interpersonal relatedness. From Blatt's perspective, mental health is characterised by the relative balance between these two developmental capacities, whereas mental illness is characterised by an overinvestment in one capacity and a defensive avoidance of the other, leading to qualitatively distinct forms of personality disorder (e.g., introjective vs. anaclitic forms; Blatt and Luyten 2009; Blatt and Shichman 1983; Blatt and Zuroff 1992; Luyten and Blatt 2013). Based on this theoretical outline, my argument is that ‘personality functioning’, from Blatt's viewpoint, cannot be understood as a general latent factor of these (and related) personality capacities; instead, personality functioning from Blatt's viewpoint can be more appropriately understood in terms of two coevolving, yet distinct, capacities.

To illustrate, if one were to reoperationalise the current construct of personality functioning from Blatt's viewpoint, one would not simply ‘explain away’ the various self‐related and interpersonal‐related difficulties with a general factor of personality (as most existing empirical practices typically operate). Instead, one would likely use sophisticated, state‐space models to conceptualise personality functioning as a dynamic system that evolves from a dialectic interaction between two capacities: a ‘self‐focused’ capacity (that appears to be primarily linked to neurotic and behavioural difficulties) and an ‘other‐focused’ capacity (that appears to be primarily linked to interpersonal difficulties) (see Zavlis 2026b for an empirical test of this viewpoint). Indeed, as recent computer simulations indicate, personality functioning can be theoretically understood in this rich and two‐dimensional way: that is, in terms of two *discrete latent factors* indicating a persistent occupation with the self or a persistent occupation with others or a healthy balance of the two (Zavlis et al. 2026). If personality functioning was to be conceptualised in Blatt's dialectic manner, then it would not be viewed as a single latent entity but as a dynamic entity that either maladaptively fixates or adaptively balances self‐functioning and interpersonal functioning.

Finally, we reach Fonagy and colleagues' mentalising model of personality (Fonagy and Allison 2023; Fonagy and Luyten 2009, 2018; Fonagy et al. 2015, 2017, 2018; Fonagy and Target 1997). As outlined previously, this model implies that personality development is intimately dependent on the development of ‘mentalising’: that is, the capacity to experience oneself and others as intentional agents with distinct thoughts, feelings and wishes (Fonagy et al. 2018). Importantly, mentalising is both multidimensional (as it involves various distinct processes) and contextual (as it differs fundamentally across relational contexts) (Hawksdale et al. 2025). Based on these considerations, my argument is that ‘personality functioning’, from a mentalising perspective, cannot be understood as a general latent factor of self–interpersonal functions; instead, personality functioning from this perspective can be more clearly understood as relational functioning: that is, as the capacity to reflect on self–other mental states in a balanced way across relationships (Zavlis et al. 2026).

To illustrate, if one were to operationalise personality functioning from a mentalising viewpoint, one would not extract a single severity dimension from a heterogeneous set of self–other personality indicators (Hawksdale et al. 2025). Instead, one would likely create a multidimensional measure of self–other functioning that captures distinct aspects of ‘mental’ or ‘relational’ inference—including, but not limited to, the extent to which self–other understanding is automatic versus controlled, cognitive versus affective, and internally versus externally focused (Hawksdale et al. 2025). Importantly, such a measure would also separate how personality ‘functions’ across different relational contexts, as mentalising (aka ‘self–other understanding’) is known to differ fundamentally across relationships (e.g., child–parent vs. peer relationships) (Zavlis 2024; Zavlis et al. 2026; Zavlis and Fonagy 2024a). Thus, from a mentalising perspective, personality functioning could never be coherently reduced to a general factor of ‘personality pathology’ because its impairment could never be divorced from specific relational contexts or be framed as a unitary deficit that occurs invariantly across such contexts.

In summary, the above outline suggests that personality functioning can be operationalised in at least three distinct ways: namely, in a configurational way (that distils personality functioning in three distinct mixture distributions, only one of which is relevant for defining personality pathology), a dialectical way (that casts personality functioning as the relative balance or imbalance of self‐functioning and interpersonal functioning) and a relational way (that defines personality functioning as a process of meaning‐making within specific relationships). Together, these perspectives suggest that there is no basis for treating personality functioning as a fixed theoretical entity because there exist at least three plausible theoretical alternatives (each with a considerable amount of evidence base). In the next section, I conclude the paper by suggesting that the field of personality disorder can only progress by embracing this form of theoretical pluralism.

## 5 Discussion

In this paper, I have argued that the current conception of personality functioning as a unitary latent entity is neither theoretically exclusive nor supported by any of the major psychodynamic theories typically invoked to justify it. At first, I reviewed major psychodynamic perspectives on personality, showing that they all conceptualise personality (and its ‘functioning’) in radically different ways. Subsequently, I showed how the existing operationalisation of personality functioning is fundamentally incompatible with these psychodynamic perspectives. Finally, I argued that personality functioning can be operationalised in at least three distinct ways that follow directly from seminal theories on this topic.

Although these arguments are cogent and evidence‐based, they may still be challenged by at least two counterpoints. A first counterpoint might involve the empirical finding that personality disorder indicators form a general ‘personality severity’ factor (Sharp et al. 2015). Indeed, given that this observation has been replicated across diverse samples, measures and statistical analyses, it may be argued that personality dysfunction can only be modelled in this unitary ‘general factor’ way (Hopwood et al. 2025; Kerber et al. 2024; Ohse et al. 2025).

However, although this counterpoint is empirically interesting, the level on which it operates is fundamentally distinct from the one I have focused on in this essay. That is, as outlined earlier, the level on which this finding operates is fundamentally descriptive, as all it implies is that issues from one domain of functioning tend to co‐vary with issues from other domains of functioning, such that all of them can be represented with a general summary index. On its own, therefore, this descriptive pattern cannot be employed as evidence that personality functioning is ‘unitary’ because the level on which it operates is simply descriptive, not explanatory or mechanistic (i.e., this factor may exist, but it can only be interpreted as a descriptive severity index, not a substantive theoretical process; see Watts et al. 2023).

Still, as we have seen previously, the assumption that a ‘general personality factor’ underpins the universe of personality disorder indicators may, under certain ontological commitments, be interpreted in an explanatory manner (e.g., Borsboom et al. 2003). Indeed, as some theorists have suggested, personality functioning can be interpreted as an explanatory general factor that makes personality traits change over time in a coordinated way (Hopwood et al. 2025). Likewise, as other theorists have implied, personality functioning may also be interpreted as an explanatory general factor that confers liability to all psychopathology, not just personality pathology (e.g., see Kerber et al. 2024). From these perspectives, therefore, personality functioning is not simply a descriptive factor, reflecting ‘personality severity’; instead, personality functioning is a unitary process that ‘mediates all types of psychopathology’ (see Bender 2019, 356).

Based on this, an alternative counterpoint to the present essay might be that the general factor of personality pathology can be interpreted substantively and is currently the best possible representation of personality pathology. Alas, however, although ‘personality functioning’ may be interpreted as a theoretical general factor, the theoretical (not descriptive) status of such a factor is only weakly supported by available evidence for at least three reasons. First and foremost, as various psychometricians and philosophers of science have long noted, general factor models carry with them a host of ontological commitments that appear to be unrealistic when it comes to the nature of psychological constructs (e.g., the assumption that a unitary latent entity causes variation in various observed variables, something that is increasingly discredited by both psychological and neurobiological ‘systems research’; Barrett and Campbell 2017; Borsboom 2005; Borsboom et al. 2003, 2019; Dolan and Borsboom 2023; Revelle 2024; Strickland and Johnson 2021; Touroutoglou et al. 2015; van Bork et al. 2017; Van Der Maas et al. 2006).

Second, even if we accepted these ontological commitments, a key and underappreciated issue with the general factor interpretation of personality functioning is that it leads to at least one paradoxical, yet logical, conclusion regarding the nature of psychopathology: namely, the view of all ‘mental’ disorders as ‘personality’ disorders, because all of them are relatively equally defined by the general factors of personality and psychopathology (see Zavlis and Fonagy 2024b, 2026 for more details).

Finally, even if we were to set aside all of these statistical and theoretical problems, one of the most important shortcomings of the literature on personality functioning is that it has not subjected the general factor models of personality functioning to genuine empirical risk because it has not examined them vis‐à‐vis alternative statistical models that may explain the data equally well, if not better—for instance, network models (Borsboom 2017), more recent generative models (Zavlis et al. 2026) or even, at the very least, alternative latent variable models (like correlated factor models rather than general factor models; Dolan and Borsboom 2023; Zavlis 2025a). Indeed, given that reanalyses of existing datasets suggest that alternative models could fit the data just as well (and, in some cases, even better; e.g., see Zavlis 2025a, 2026a, 2026b, 2026c), any evidence in favour of general factor models needs to be contextualised against such counter‐evidence (otherwise, the field risks reifying a general factor construct whose empirical status remains questionable, at best, or completely illusory, at worst; see Fried 2020; Revelle 2024; Watts et al. 2023).

Together, these points lead me to the final conclusion I would like to draw in this piece: namely, that progress in this field can only be achieved when researchers stop reifying the concept of personality functioning (by interpreting and modelling it in only one way) and start instead examining it in multiple different ways. Indeed, as philosophers of science have long noted, scientific fields can only mature when they examine multiple competing hypotheses in a genuinely critical, risky and self‐judgemental way (e.g., see Platt 1964; Popper 1959). If the field of personality pathology wishes to shy away from this basic scientific principle (by continuing to define personality functioning in only one way, computing the same general factor summaries and never actually examining whether alternative theoretical models may fit personality data equally well, if not better), then the field is unlikely to converge into an ontologically accurate view of personality because it will have simply ossified it into a particular theoretical viewpoint. If the field, however, wishes to genuinely advance the science of personality (and its functioning), then it must embrace theoretical pluralism by, first, acknowledging that there are multiple competing theories of personality (functioning) and, second, critically examining each in order to find out which one, if any, is right.^1^

## Funding

The author has nothing to report.

## Data Availability

Data sharing is not applicable to this article as no datasets were generated or analysed during the current study.

## Endnotes

^1^
Here, I say ‘if any’ because it may well be the case that no theory on ‘personality functioning’ turns out to be right. In that case, the field might wish to move towards a more descriptive (and arguably more objective) classification system (akin to the one already embraced by ICD‐11) whereby personality function and dysfunction are defined descriptively as a general summary of personality disorder severity (see Tyrer et al. 2011, 2019). Indeed, one could very well make the case that the best path forward is to abandon all of our rarefied theories on ‘what it means to be human’ (Bender 2019; Sharp and Wall 2021) and simply look at the actual patients suffering from personality‐related problems, hear their perspectives on the kinds of difficulties they face and build a classification system of those difficulties from the bottom‐up rather than from the (dogmatically imposed) top‐down. Such an approach would be consistent with broader calls for patient‐led research in psychopathology (Meyer‐Lindenberg and Lipinski 2025) and is likely to lead to a more clinically meaningful (and less esoteric) understanding of personality and its pathology (e.g., Zavlis, Fonagy, and Luyten 2025). However, I find it quite unlikely that such an approach will be embraced anytime soon, given that the field seems more preoccupied with promoting ideological perspectives on the topic as opposed to critically evaluating those perspectives against either alternative theories or what patients with lived experience have to say about this topic.

## References

[pmh70085-bib-0001] Barrett, L. F. , and C. Campbell . 2017. How Emotions Are Made: The Secret Life of the Brain. Brilliance Audio.

[pmh70085-bib-0002] Bender, D. S. 2019. “The *p*‐Factor and What It Means to Be Human: Commentary on Criterion A of the AMPD in HiTOP.” Journal of Personality Assessment 101, no. 4: 356–359. 10.1080/00223891.2018.1492928. 30160537

[pmh70085-bib-0003] Bender, D. S. , L. C. Morey , and A. E. Skodol . 2014. “Toward a Model for Assessing Level of Personality Functioning in DSM‐5, Part I: A Review of Theory and Methods.” In Personality Assessment in the DSM‐5, 35–49. Routledge. 10.1080/00223891.2011.583808 22804672

[pmh70085-bib-0004] Bion, W. R. 1962. Learning From Experience. Heinemann.

[pmh70085-bib-0005] Blatt, S. 1992. “The Differential Effect of Psychotherapy and Psychoanalysis With Anaclitic and Introjective Patients: The Menninger Psychotherapy Research Project Revisited.” Journal of the American Psychoanalytic Association 40, no. 3: 691–724. 10.1177/000306519204000303. 1401718

[pmh70085-bib-0006] Blatt, S. J. 1995. “The Destructiveness of Perfectionism: Implications for the Treatment of Depression.” American Psychologist 50, no. 12: 1003–1020. 8561378 10.1037//0003-066x.50.12.1003

[pmh70085-bib-0007] Blatt, S. J. 2008. Polarities of Experience. American Psychological Association.

[pmh70085-bib-0008] Blatt, S. J. , and P. Luyten . 2009. “A Structural–Developmental Psychodynamic Approach to Psychopathology: Two Polarities of Experience Across the Life Span.” Development and Psychopathology 21, no. 3: 793–814. 19583884 10.1017/S0954579409000431

[pmh70085-bib-0009] Blatt, S. J. , and S. Shichman . 1983. “Two Primary Configurations of Psychopathology.” Psychoanalysis & Contemporary Thought 6, no. 2: 187–254.

[pmh70085-bib-0010] Blatt, S. J. , and D. C. Zuroff . 1992. “Interpersonal Relatedness and Self‐Definition: Two Prototypes for Depression.” Clinical Psychology Review 12, no. 5: 527–562.

[pmh70085-bib-0011] Borsboom, D. 2005. Measuring the Mind: Conceptual Issues in Contemporary Psychometrics. Cambridge University Press.

[pmh70085-bib-0012] Borsboom, D. 2017. “A Network Theory of Mental Disorders.” World Psychiatry 16, no. 1: 5–13. 10.1002/wps.20375. 28127906 PMC5269502

[pmh70085-bib-0013] Borsboom, D. , A. O. J. Cramer , and A. Kalis . 2019. “Reductionism in Retreat.” Behavioral and Brain Sciences 42: e32. 10.1017/S0140525X18002091. 30940245

[pmh70085-bib-0014] Borsboom, D. , G. J. Mellenbergh , and J. Van Heerden . 2003. “The Theoretical Status of Latent Variables.” Psychological Review 110, no. 2: 203–219. 10.1037/0033-295X.110.2.203. 12747522

[pmh70085-bib-0015] Dolan, C. V. , and D. Borsboom . 2023. “Interpretational Issues With the Bifactor Model: A Commentary on ‘Defining the *p*‐Factor: An Empirical Test of Five Leading Theories’ by Southward, Cheavens, and Coccaro.” Psychological Medicine 53, no. 7: 2744–2747. 37039112 10.1017/S0033291723000533

[pmh70085-bib-0016] Erikson, E. H. 1963. Childhood and Society. Norton.

[pmh70085-bib-0017] Fairbairn, W. R. 1941. “A Revised Psychopathology of the Psychoses and Psychoneuroses.” In Psychoanalytic Studies of the Personality. Routledge.

[pmh70085-bib-0018] Fairbairn, W. R. D. 1952. Psychoanalytic Studies of the Personality. Routledge & CRC Press. https://www.routledge.com/Psychoanalytic‐Studies‐of‐the‐Personality/Fairbairn/p/book/9780415107372.

[pmh70085-bib-0019] Fairbairn, W. R. D. 1954. An Object‐Relations Theory of the Personality. Basic Books.

[pmh70085-bib-0020] Fonagy, P. , and E. Allison . 2023. “Beyond Mentalizing: Epistemic Trust and the Transmission of Culture.” Psychoanalytic Quarterly 92, no. 4: 599–640. 38095858 10.1080/00332828.2023.2290023

[pmh70085-bib-0021] Fonagy, P. , G. Gergely , and E. L. Jurist . 2018. Affect Regulation, Mentalization and the Development of the Self. Routledge.

[pmh70085-bib-0022] Fonagy, P. , and P. Luyten . 2009. “A Developmental, Mentalization‐Based Approach to the Understanding and Treatment of Borderline Personality Disorder.” Development and Psychopathology 21, no. 4: 1355–1381. 19825272 10.1017/S0954579409990198

[pmh70085-bib-0023] Fonagy, P. , and P. Luyten . 2018. “Attachment, Mentalizing, and the Self.” In Handbook of Personality Disorders: Theory, Research, and Treatment, vol. 2, 123–140. Guilford Press.

[pmh70085-bib-0024] Fonagy, P. , P. Luyten , and E. Allison . 2015. “Epistemic Petrification and the Restoration of Epistemic Trust: A New Conceptualization of Borderline Personality Disorder and Its Psychosocial Treatment.” Journal of Personality Disorders 29, no. 5: 575–609. 10.1521/pedi.2015.29.5.575. 26393477

[pmh70085-bib-0025] Fonagy, P. , P. Luyten , E. Allison , and C. Campbell . 2017. “What We Have Changed Our Minds About: Part 1. Borderline Personality Disorder as a Limitation of Resilience.” Borderline Personality Disorder and Emotion Dysregulation 4, no. 1: 11. 10.1186/s40479-017-0061-9. 28413687 PMC5389119

[pmh70085-bib-0026] Fonagy, P. , and M. Target . 1997. “Attachment and Reflective Function: Their Role in Self‐Organization.” Development and Psychopathology 9, no. 4: 679–700. 10.1017/S0954579497001399. 9449001

[pmh70085-bib-0027] Freud, A. 1966. The Ego and the Mechanisms of Defence (The Writings of Anna Freud). International Universities Press. (Original Work Published 1936).

[pmh70085-bib-0028] Freud, S. 1953. “Three Essays on the Theory of Sexuality.” In The Standard Edition of the Complete Psychological Works of Sigmund Freud, Vol. 7, edited by J. Strachey (Ed. & Trans.). Hogarth Press. (Original Work Published 1905)

[pmh70085-bib-0029] Freud, S. 1957. “Introductory Lectures on Psycho‐Analysis.” In The Standard Edition of the Complete Psychological Works of Sigmund Freud, Vols. XV & XVI, edited by J. Strachey (Ed. & Trans.). Hogarth Press. (Original Work Published 1917)

[pmh70085-bib-0030] Freud, S. 1959. “Inhibitions, Symptoms, and Anxiety.” In The Standard Edition of the Complete Psychological Works of Sigmund Freud, Vol. 20, edited by J. Strachey (Ed. & Trans.). Hogarth Press. (Original Work Published 1926)

[pmh70085-bib-0031] Freud, S. 1961. “The Ego and the Id.” In Strachey, J. et al (Trans.).” In The Standard Edition of the Complete Psychological Works of Sigmund Freud, Vol. 19. Hogarth Press. (Original Work Published 1923)

[pmh70085-bib-0032] Fried, E. I. 2020. “Lack of Theory Building and Testing Impedes Progress in the Factor and Network Literature.” Psychological Inquiry 31, no. 4: 271–288. 10.1080/1047840X.2020.1853461.

[pmh70085-bib-0033] Fried, E. I. , A. L. Greene , and N. R. Eaton . 2021. “The *p* Factor Is the Sum of Its Parts, for Now.” World Psychiatry 20, no. 1: 69–70. 33432741 10.1002/wps.20814 PMC7801826

[pmh70085-bib-0034] García, L. F. , F. Gutiérrez , O. García , and A. Aluja . 2024. “The Alternative Model of Personality Disorders: Assessment, Convergent and Discriminant Validity, and a Look to the Future.” Annual Review of Clinical Psychology 20: 431–455. 10.1146/annurev-clinpsy-081122-010709 38211624

[pmh70085-bib-0035] Gilbert, K. J. 2026. “Criterion A as Developmental Severity: Reclaiming the Psychodynamic Core of Personality Functioning.” Personality and Mental Health 20, no. 1: e70059. 41451831 10.1002/pmh.70059

[pmh70085-bib-0036] Hartmann, H. 1958. Ego Psychology and the Problem of Adaptation. International Universities Press.

[pmh70085-bib-0037] Hartmann, H. , E. Kris , and R. M. Loewenstein . 1947. “Notes on the Theory of Aggression.” Psychoanalytic Study of the Child 3, no. 1: 9–36.

[pmh70085-bib-0038] Hawksdale, H. , B. Meng , J. Feigenbaum , P. Fonagy , and T. Nolte . 2025. “Measurement of Mentalizing: A Systematic Review and Development of a Construct Validity Framework.” Clinical Psychology Review 122: 102657. 10.1016/j.cpr.2025.102657. 41109035

[pmh70085-bib-0039] Hopwood, C. J. 2025. “Commentary on Kerber et al. (2025): Personality Functioning Only Makes Sense From a Psychodynamic Perspective.” Zeitschrift für Klinische Psychologie und Psychotherapie 54, no. 3: 166–168. 10.1026/1616-3443/a000824.

[pmh70085-bib-0040] Hopwood, C. J. , C. C. Driver , L. C. Morey , and A. E. Skodol . 2025. “Personality Functioning as Generalized Correlated Changes in Personality Traits.” Journal of Psychopathology and Clinical Science 135, no. 2: 297–305. 41066267 10.1037/abn0001065

[pmh70085-bib-0041] Kaiser, T. 2026. “Toward Mechanistic Clarity in Personality Functioning: A Behavioral Account of the Self.” Journal of Psychopathology and Clinical Science 135: 343–344. 10.1037/abn0001105. 41785140

[pmh70085-bib-0042] Kerber, A. , J. C. Ehrenthal , J. Zimmermann , et al. 2024. “Examining the Role of Personality Functioning in a Hierarchical Taxonomy of Psychopathology Using Two Years of Ambulatory Assessed Data.” Translational Psychiatry 14, no. 1: 340. 39181872 10.1038/s41398-024-03046-z PMC11344763

[pmh70085-bib-0043] Kernberg, O. 1967. “Borderline Personality Organization.” Journal of the American Psychoanalytic Association 15, no. 3: 641–685. 10.1177/000306516701500309. 4861171

[pmh70085-bib-0044] Kernberg, O. F. 1970. “A Psychoanalytic Classification of Character Pathology.” Journal of the American Psychoanalytic Association 18, no. 4: 800–822. 5493243 10.1177/000306517001800403

[pmh70085-bib-0045] Kernberg, O. F. 1975. Borderline Conditions and Pathological Narcissism. Jason Aronson.

[pmh70085-bib-0046] Kernberg, O. F. 1976. Object Relations Theory and Clinical Psychoanalysis. Jason Aronson.

[pmh70085-bib-0047] Kernberg, O. F. 1984. Severe Personality Disorders: Psychotherapeutic Strategies. Yale University.

[pmh70085-bib-0048] Kernberg, O. F. 2016. “What Is Personality?” Journal of Personality Disorders 30, no. 2: 145–156. 27027422 10.1521/pedi.2106.30.2.145

[pmh70085-bib-0049] Klein, M. 1940. “Mourning and Its Relation to Manic‐Depressive States.” International Journal of Psychoanalysis 21, no. 1: 125–153.

[pmh70085-bib-0050] Klein, M. 1946. “Notes on Some Schizoid Mechanisms.” International Journal of Psychoanalysis 27: 99–110. 20261821

[pmh70085-bib-0051] Laplanche, J. , and J.‐B. Pontalis . 2018. The Language of Psychoanalysis. Routledge. 10.4324/9780429482243.

[pmh70085-bib-0052] Loevinger, J. 1976. Ego Development: Conceptions and Theories. Jossey‐Bass.

[pmh70085-bib-0053] Luyten, P. , and S. J. Blatt . 2013. “Interpersonal Relatedness and Self‐Definition in Normal and Disrupted Personality Development: Retrospect and Prospect.” American Psychologist 68, no. 3: 172–183. 23586492 10.1037/a0032243

[pmh70085-bib-0054] Luyten, P. , C. Campbell , E. Allison , and P. Fonagy . 2020. “The Mentalizing Approach to Psychopathology: State of the Art and Future Directions.” Annual Review of Clinical Psychology 16, no. 1: 297–325. 10.1146/annurev-clinpsy-071919-015355 32023093

[pmh70085-bib-0055] Luyten, P. , and P. Fonagy . 2015. “The Neurobiology of Mentalizing.” Personality Disorders: Theory, Research, and Treatment 6, no. 4: 366–379. 10.1037/per0000117 26436580

[pmh70085-bib-0056] Luyten, P. , B. Lowyck , and S. J. Blatt . 2017. “Mechanisms of Change Through the Lens of Two‐Polarities Models of Personality Development: State of the Art and New Directions.” Psychoanalytic Inquiry 37, no. 3: 179–190.

[pmh70085-bib-0057] MacWilliams, N. 2020. Psychoanalytic Diagnosis: Understanding Personality Structure in the Clinical Process. 2nd ed. Guilford Press.

[pmh70085-bib-0058] Mahler, M. S. , F. Pine , and A. Bergman . 1975. The Psychological Birth of the Human Infant: Symbiosis and Individuation. Basic Books. 4445412

[pmh70085-bib-0059] McCabe, G. A. , J. R. Oltmanns , and T. A. Widiger . 2022. “The General Factors of Personality Disorder, Psychopathology, and Personality.” Journal of Personality Disorders 36, no. 2: 129–156. 10.1521/pedi_2021_35_530. 34287069

[pmh70085-bib-0060] Meyer‐Lindenberg, A. , and S. Lipinski . 2025. “Involving Lived Experience Experts in Translational Research.” Nature Reviews Psychology 4, no. 3: 149–150. 10.1038/s44159-025-00424-2.

[pmh70085-bib-0061] Morey, L. C. 2019. “Thoughts on the Assessment of the DSM–5 Alternative Model for Personality Disorders: Comment on Sleep et al. (2019).” Psychological Assessment 31: 1192–1199. 31580132 10.1037/pas0000710

[pmh70085-bib-0062] Morey, L. C. , M. N. McCredie , D. S. Bender , and A. E. Skodol . 2022. “Criterion A: Level of Personality Functioning in the Alternative DSM–5 Model for Personality Disorders.” Personality Disorders: Theory, Research, and Treatment 13, no. 4: 305–315. 10.1037/per0000551 35787111

[pmh70085-bib-0063] Ohse, L. , A. Kerber , J. Zimmermann , et al. 2025. “Empirical Convergence Between Kernberg's Model of Personality Organization and the Alternative DSM‐5 Model for Personality Disorders.” Personality and Mental Health 19, no. 1: e1641. 10.1002/pmh.1641. 39610209

[pmh70085-bib-0064] Oltmanns, J. R. , G. T. Smith , T. F. Oltmanns , and T. A. Widiger . 2018. “General Factors of Psychopathology, Personality, and Personality Disorder: Across Domain Comparisons.” Clinical Psychological Science 6, no. 4: 581–589. 10.1177/2167702617750150. 30221082 PMC6132273

[pmh70085-bib-0065] Pine, F. 1990. Drive, Ego, Object, and Self: A Synthesis for Clinical Work. Perseus.

[pmh70085-bib-0066] Platt, J. R. 1964. “Strong Inference: Certain Systematic Methods of Scientific Thinking May Produce Much More Rapid Progress Than Others.” Science 146, no. 3642: 347–353. 17739513 10.1126/science.146.3642.347

[pmh70085-bib-0067] Popper, K. 1959. The Logic of Scientific Discovery. Basic Books.

[pmh70085-bib-0068] Rapaport, D. 1951. Organization and Pathology of Thought. Columbia University Press.

[pmh70085-bib-0069] Revelle, W. 2024. “The Seductive Beauty of Latent Variable Models: Or Why I Don't Believe in the Easter Bunny.” Personality and Individual Differences 221: 112552.

[pmh70085-bib-0070] Sharp, C. 2022. “Fulfilling the Promise of the LPF: Comment on Morey et al. (2022).” Personality Disorders: Theory, Research, and Treatment 13: 316–320. 10.1037/per0000567 35787112

[pmh70085-bib-0071] Sharp, C. , and K. Wall . 2021. “DSM‐5 Level of Personality Functioning: Refocusing Personality Disorder on What It Means to Be Human.” Annual Review of Clinical Psychology 17: 313–337. 10.1146/annurev-clinpsy-081219-105402 33306924

[pmh70085-bib-0072] Sharp, C. , A. G. Wright , J. C. Fowler , et al. 2015. “The Structure of Personality Pathology: Both General (‘g’) and Specific (‘s’) Factors?” Journal of Abnormal Psychology 124, no. 2: 387–398. 25730515 10.1037/abn0000033

[pmh70085-bib-0073] Shedler, J. 2022. “The Personality Syndromes.” In Personality Disorders, 3–32. Oxford University Press.

[pmh70085-bib-0074] Shedler, J. , and D. Westen . 2004. “Dimensions of Personality Pathology: An Alternative to the Five‐Factor Model.” American Journal of Psychiatry 161, no. 10: 1743–1754. 15465966 10.1176/ajp.161.10.1743

[pmh70085-bib-0075] Sleep, C. E. , and D. R. Lynam . 2022. “The Problems With Criterion A: A Comment on Morey et al. (2022).” Personality Disorders: Theory, Research, and Treatment 13: 325–327. 10.1037/per0000585 35787114

[pmh70085-bib-0076] Sleep, C. E. , D. R. Lynam , T. A. Widiger , M. L. Crowe , and J. D. Miller . 2019. “Difficulties With the Conceptualization and Assessment of Criterion A in the DSM–5 Alternative Model of Personality Disorder: A Reply to Morey (2019).” Psychological Assessment 31: 1200–1205. 31580133 10.1037/pas0000758

[pmh70085-bib-0077] Sleep, C. E. , B. Weiss , D. R. Lynam , and J. D. Miller . 2020. “The DSM–5 Section III Personality Disorder Criterion A in Relation to Both Pathological and General Personality Traits.” Personality Disorders: Theory, Research, and Treatment 11, no. 3: 202–212. 10.1037/per0000383 31804130

[pmh70085-bib-0078] Strickland, J. C. , and M. W. Johnson . 2021. “Rejecting Impulsivity as a Psychological Construct: A Theoretical, Empirical, and Sociocultural Argument.” Psychological Review 128, no. 2: 336–361. 32969672 10.1037/rev0000263 PMC8610097

[pmh70085-bib-0079] Touroutoglou, A. , K. A. Lindquist , B. C. Dickerson , and L. F. Barrett . 2015. “Intrinsic Connectivity in the Human Brain Does Not Reveal Networks for ‘Basic’ Emotions.” Social Cognitive and Affective Neuroscience 10, no. 9: 1257–1265. 10.1093/scan/nsv013. 25680990 PMC4560947

[pmh70085-bib-0080] Tyrer, P. , M. Crawford , R. Mulder , et al. 2011. “The Rationale for the Reclassification of Personality Disorder in the 11th Revision of the International Classification of Diseases (ICD‐11).” Personality and Mental Health 5, no. 4: 246–259. 10.1002/pmh.190.

[pmh70085-bib-0081] Tyrer, P. , R. Mulder , Y.‐R. Kim , and M. J. Crawford . 2019. “The Development of the ICD‐11 Classification of Personality Disorders: An Amalgam of Science, Pragmatism, and Politics.” Annual Review of Clinical Psychology 15: 481–502. 10.1146/annurev-clinpsy-050718-095736 30601688

[pmh70085-bib-0082] Vaillant, G. E. 1992. Ego Mechanisms of Defense: A Guide for Clinicians and Researchers. American Psychiatric Pub.

[pmh70085-bib-0083] Vaillant, G. E. 2012. Adaptation to Life. Harvard University Press.

[pmh70085-bib-0084] van Bork, R. , S. Epskamp , M. Rhemtulla , D. Borsboom , and H. L. J. van der Maas . 2017. “What Is the *p*‐Factor of Psychopathology? Some Risks of General Factor Modeling.” Theory & Psychology 27: 759–773. 10.1177/095935431773718.

[pmh70085-bib-0085] Van Der Maas, H. L. , C. V. Dolan , R. P. Grasman , J. M. Wicherts , H. M. Huizenga , and M. E. Raijmakers . 2006. “A Dynamical Model of General Intelligence: The Positive Manifold of Intelligence by Mutualism.” Psychological Review 113, no. 4: 842–861. 17014305 10.1037/0033-295X.113.4.842

[pmh70085-bib-0086] Watts, A. L. , A. L. Greene , W. Bonifay , and E. I. Fried . 2023. “A Critical Evaluation of the *p*‐Factor Literature.” Nature Reviews Psychology 3: 1–122.

[pmh70085-bib-0087] Widiger, T. A. 2011. “Personality and Psychopathology.” World Psychiatry 10, no. 2: 103–106. 21633679 10.1002/j.2051-5545.2011.tb00024.x PMC3104878

[pmh70085-bib-0088] Widiger, T. A. , B. Bach , M. Chmielewski , et al. 2019. “Criterion A of the AMPD in HiTOP.” Journal of Personality Assessment 101, no. 4: 345–355. 29746190 10.1080/00223891.2018.1465431

[pmh70085-bib-0089] Widiger, T. A. , and M. M. Smith . 2025. “Personality Disorders: Current Conceptualizations and Challenges.” Annual Review of Clinical Psychology 21, no. 1: 169–192. 10.1146/annurev-clinpsy-081423-030513. 39836877

[pmh70085-bib-0090] Winnicott, D. W. 1951. Transitional Objects and Transitional Phenomena. Tavistock.

[pmh70085-bib-0091] Winnicott, D. W. 1960. “The Theory of the Parent‐Infant Relationship.” International Journal of Psycho‐Analysis 41: 585–595. 13785877

[pmh70085-bib-0092] Winnicott, D. W. 1965. The Maturational Processes and the Facilitating Environment: Studies in the Theory of Emotional Development, 295. International Universities Press.

[pmh70085-bib-0093] Zavlis, O. 2024. The Illusion of Personality: Why Personality Disorders Are Actually Relational Disorders. PsyArXiv. 10.31234/osf.io/b4d6v.

[pmh70085-bib-0094] Zavlis, O. 2025a. “Personality Functioning Is Not a Unitary Entity.” Personality and Mental Health 19, no. 3: e70033. 10.1002/pmh.70033. 40583122 PMC12206940

[pmh70085-bib-0095] Zavlis, O. 2025b. The Idea of Personality Functioning Should Be Critically Examined, Not Reified: A Reply to Zimmermann et al. (2025). PsyArXiv. 10.31234/osf.io/gpwef_v1.

[pmh70085-bib-0096] Zavlis, O. 2026a. A Critical Examination of the General Factor of Personality Change. PsyArXiv. 10.31234/osf.io/mf3r6_v1.

[pmh70085-bib-0097] Zavlis, O. 2026b. Formalising and Testing Kernberg's Theory of Personality Organisation. PsyArXiv. 10.31234/osf.io/kq5dg_v1.

[pmh70085-bib-0098] Zavlis, O. 2026c. Is Personality Functioning Unitary or Dialectic? A Dynamical Systems Analysis. PsyArXiv. 10.31234/osf.io/4nezv_v1.

[pmh70085-bib-0099] Zavlis, O. , and P. Fonagy . 2024a. “Beyond Descriptive Models of Personality Problems.” Journal of Personality Assessment 107: 164–167. 10.1080/00223891.2024.2430322. 39576884

[pmh70085-bib-0100] Zavlis, O. , and P. Fonagy . 2024b. Either All Mental Disorders Are Personality Disorders or There Are No Personality Disorders. OSF. 10.31234/osf.io/4xpdg.

[pmh70085-bib-0101] Zavlis, O. , and P. Fonagy . 2026. The Construct ‘Personality Functioning’ Is Currently Not Logically Defined. PsyArXiv. 10.31234/osf.io/zncr7_v1.

[pmh70085-bib-0102] Zavlis, O. , P. Fonagy , and P. Luyten . 2025. “The Most Important Aims of Psychotherapy: To Love, to Work, and to Find Meaning.” Lancet Psychiatry 12, no. 3: 173–174. 10.1016/S2215-0366(25)00006-9. 39978979

[pmh70085-bib-0103] Zavlis, O. , M. Moutoussis , P. Fonagy , and G. Story . 2026. “A Generative Model of Personality Disorder as a Relational Disorder.” Journal of Psychopathology and Clinical Science 135, no. 1: 25–46. 10.1037/abn0001010. 40705631

[pmh70085-bib-0104] Zavlis, O. , G. Story , C. Friedrich , P. Fonagy , and M. Moutoussis . 2025. “A Systematic Review of Computational Modeling of Interpersonal Dynamics in Psychopathology.” Nature Mental Health 3, no. 8: 932–942. 10.1038/s44220-025-00465-9.

[pmh70085-bib-0105] Zimmermann, J. , A. Kerber , S. Hörz‐Sagstetter , C. J. Hopwood , and L. Ohse . 2025. “The Total Score of the Level of Personality Functioning Scale Is Empirically and Theoretically Well‐Justified: A Reply to Zavlis (2025).” Personality and Mental Health 19, no. 4: 1–3. 10.1002/pmh.70049. PMC12589701 41194305

